# Identification of quantitative trait loci and candidate genes for grain superoxide dismutase activity in wheat

**DOI:** 10.1186/s12870-024-05367-z

**Published:** 2024-07-27

**Authors:** Kejia Qu, Jiqing Wang, Yukun Cheng, Bin Bai, Xianchun Xia, Hongwei Geng

**Affiliations:** 1 College of Agriculture, The Engineering and Technology Research Center for High-quality, Xinjiang Agricultural University, Urumqi, 830052 China; 2 Wheat Research Institute, Gansu Academy of Agricultural Sciences, Lanzhou, 730070 China; 3 Institute of Crop Sciences, National Wheat Improvement Center, Chinese Academy of Agricultural Sciences (CAAS), Beijing, 100081 China

**Keywords:** Candidate gene, Quantitative trait locus (QTL), Superoxide dismutase (SOD) activity, Wheat

## Abstract

**Background:**

Superoxide dismutase (SOD) can greatly scavenge reactive oxygen species (ROS) in plants. SOD activity is highly related to plant stress tolerance that can be improved by overexpression of SOD genes. Identification of SOD activity-related loci and potential candidate genes is essential for improvement of grain quality in wheat breeding. However, the loci and candidate genes for relating SOD in wheat grains are largely unknown. In the present study, grain SOD activities of 309 recombinant inbred lines (RILs) derived from the ‘Berkut’ × ‘Worrakatta’ cross were assayed by photoreduction method with nitro-blue tetrazolium (NBT) in four environments. Quantitative trait loci (QTL) of SOD activity were identified using inclusive composite interval mapping (ICIM) with the genotypic data of 50 K single nucleotide polymorphism (SNP) array.

**Results:**

Six QTL for SOD activity were mapped on chromosomes 1BL, 4DS, 5AL (2), and 5DL (2), respectively, explaining 2.2 ~ 7.4% of the phenotypic variances. Moreover, *QSOD.xjau-1BL*, *QSOD.xjau-4DS*, *QSOD.xjau-5 A.1*, *QSOD.xjau-5 A.2*, and *QSOD.xjau-5DL.2* identified are likely to be new loci for SOD activity. Four candidate genes *TraesCS4D01G059500*, *TraesCS5A01G371600*, *TraesCS5D01G299900*, *TraesCS5D01G343100LC*, were identified for *QSOD.xjau-4DS*, *QSOD.xjau-5AL.1*, and *QSOD.xjau-5DL.1* (2), respectively, including three SOD genes and a gene associated with SOD activity. Based on genetic effect analysis, this can be used to identify desirable alleles and excellent allele variations in wheat cultivars.

**Conclusion:**

These candidate genes are annotated for promoting SOD production and inhibiting the accumulation of ROS during plant growth. Therefore, lines with high SOD activity identified in this study may be preferred for future wheat breeding.

**Supplementary Information:**

The online version contains supplementary material available at 10.1186/s12870-024-05367-z.

## Introduction

Wheat is an important staple food crop worldwide, providing 20.0% of calorie for humans [1, 2]. The quality of wheat-based products has gained increasing attention due to people’s growing demand for nutritious and healthy diets [3, 4]. The nutritional quality of bread can be improved through antioxidant enzyme and non-enzymatic antioxidants [5, 6]. Importantly, superoxide dismutase (SOD) plays a pivotal role in scavenging reactive oxygen species [7]. Superoxide dismutase in wheat grains can tremendously enhance dough rheological properties by reducing the production of free sulfhydryl groups [8]. Increased free sulfhydryl groups cause decreased strength of gluten network structure and flour quality [9]. Meanwhile, the elevated SOD activity contributes to improved wheat quality by increasing the content of vitamin C and vitamin E [10]. Therefore, breeding wheat varieties with high SOD activity represents a significant step forward for improvement of wheat quality.

SOD activity in wheat grain is a quantitative trait controlled by multiple genes and also influenced by environments [11]. To date, numerous QTL for SOD activity have been identified in wheat. For example, several SOD QTL were identified in wheat leaf SOD QTL on chromosomes 1B, 1D, 2 A and 6D [11]. Twenty-six SOD genes from the wheat genome were detected by bioinformatics, including Cu/Zn-SODs, Fe-SODs, and Mn-SODs [12]. These results indicate that the SOD activity of wheat is controlled by several major-effect QTL and numerous minor-effect QTL, distributing across wheat chromosomes.

SOD in wheat is associated with protecting plant cells from oxidative damage and maintaining metabolic equilibrium in organisms, but research on the correlation of SOD activity with quality traits has been rarely reported. In particular, there are few reports about SOD in wheat grains and knowledge of corresponding molecular markers, which is limiting breeding practices. There may be a risk to wheat production and resistance due to research limitations on wheat grain SOD activity. The development of functional molecular markers is important for efficient breeding. Therefore, the present study was to map the QTL for SOD activity, predict the potential candidate genes and develop molecular markers for precise marker-assisted selection, which would be served as a reference for future genetic improvement and quality breeding.

## Materials and methods

### Plant materials

Three hundred and nine F_6_ RILs developed from the cross of ‘Berkut’ × ‘Workatta’, kindly provided by Dr. Susanne Dreisigacker at the International Maize and Wheat Improvement Center (CIMMYT), were used for QTL mapping. The RILs and parental lines were grown at the Manas experimental Station of Xinjiang Academy of Agricultural Sciences in 2016, 2017, 2018 and 2019 (referred as E1, E2, E3, and E4, respectively). The experiments were conducted in randomized complete blocks with three replications, and the RILs were planted in single 2 m rows spacing in 25 cm between rows. The management of field trials was following local practices with drip irrigation. The grains of RILs were harvested at maturity, and used to extract the SOD enzyme.

### Extraction of SOD from wheat grain

The extraction of the crude SOD enzyme was performed following Wang et al. [13]. For each line, 10 g grains were milled into whole wheat flour using a 0.8 mm cyclone mill (laboratory mill 120, Perten, Swedish). The 0.1 g of whole wheat flour was immersed in 1 mL of 50 mmol/L potassium phosphate (pH 7.8) in a 2 mL Eppendorf tube, and subsequently oscillated for 1 min on a mixer oscillator. The homogenized mixture was put into an ice bath shaker for 2 h, then centrifuged at 10,000 rpm for 15 min at 4℃ (Centrifuge 5425R, Eppendorf, USA). Finally, the supernatant was collected as crude SOD enzyme extract.

### Determination of SOD activity

SOD activity was measured using the nitro-blue tetrazolium (NBT) photoreduction method [14]. Assay of each sample includes a sample tube and two control tubes. The sample tube contained 10 µL of SOD enzyme extract homogenized in a substrate composed of 150 mL of 0.05 mol/L phosphate buffer solution, 20 µL of distilled water, 30 µL of 130 mmol/L Met solution, 30 mL of 750 µmol/L NBT solution, 30 mL of 100 µmol/L EDTA-Na_2_ solution and 30 mL of 20 µmol/L solution, whereas the control tube contained the buffer without enzyme extract. One control tube was put in the darkness, while the other was placed under a 4000 lx fluorescent light for 20 min. The control tube in the dark is blank, control tube in the light is absorbance of illumination. The OD (optical density) value was determined at 560 nm using microplate reader (Synergy H1, Biotek, USA). One unit of SOD was defined as the amount of hemoglobin that inhibits the rate of NBT reduction by 50.0%. The SOD activity was calculated using the following formula:

$${\rm{SOD}}\,{\rm{activity}}\,{\rm{U\cdot}}{{\rm{g}}^{{\rm{ - 1}}}}{\rm{ = (}}{{\rm{A}}_{{\rm{ck}}}}{\rm{ - }}{{\rm{A}}_{\rm{E}}}{\rm{) \times V/(}}{{\rm{A}}_{{\rm{ck}}}}{\rm{ \times 0}}{\rm{.5 \times W \times }}{{\rm{V}}_{\rm{t}}}{\rm{)}},$$

where A_CK_ is absorbance of illumination for the control tube, A_E_ is absorbance of illumination for the sample tube, V is total sample volume (mL), V_t_ is sample volume for determination (mL) and W is sample fresh weight (g). The SOD value of each sample was measured in duplicate to ensure the accuracy of the assay. If the difference between two repeats was more than 10.0%, the experiments were carried out again.

### Statistical analysis

The data analysis was performed using Excel 2016 and SPSS 21.0. The broad-sense heritability and variance were analyzed using the QTL IciMapping V4.1 software [15]. The broad-sense heritability (*H*^*2*^) was estimated as ${H^2} = {\sigma _g}^2/({\sigma _g}^2 + {\sigma _{ge}}^2/e + {\sigma _ \in }^2/re)$, where ${{}_{g}}^{2}$ is genotypic variance, ${{}_{ge}}^{2}$ is the variance of genotype × environment interaction, ${\sigma _ \in }^2$ is residual errors, and e and rare the number of environments and replications, respectively.

### Linkage map construction

The genomic DNA was extracted from young wheat leaves using a modified cetyltrimethylammonium bromide (CTAB) method. The RILs and parents were genotyped by the wheat 50 K SNP array (the Axiom^®^ HD Wheat Genotyping Array, CapitalBio Technology Inc, Beijing). The polymorphism analysis was performed using the Genomestudiov1.0 software. A total of 11,375 polymorphic SNP markers were obtained after removing markers of distorted segregation (*P* < 0.001), monomorphic markers, and those with more than 20.0% missing data, and 1,604 bin markers were selected to construct linkage groups. The genetic linkage map was constructed with 1604 bin SNP markers, generating 28 linkage groups on 21 wheat chromosomes, spanning 2220.3 cM with an average genetic distance of 1.38 cM between two bin markers. The genetic mapping was carried out using CARTHAGENE [16].

### QTL mapping

The ICIM-ADD function in the QTL IciMapping V4.1 software was used to map QTL for the SOD activity. The LOD (logarithm of the odds) threshold was determined by 1000 × permutations, at a significance level of *P* < 0.05. The locations of QTL on wheat chromosomes were compared with the Chinese Spring (CS) genome sequence (IWGSC RefSeq v2.1) to obtain the physical positions of QTL. MapChart was used to draw genetic linkage maps [17].

### Candidate gene prediction

QTL mapping for the SOD activity was projected for further comparison. The candidate genes involved in the SOD metabolism and regulation pathways were predicted based on the physical positions of QTL flanking markers and the CS reference genome sequences. The flanking sequences of SNP markers were blasted on the NCBI website (http://www.ncbi.nlm.nih.gov/) and the CS reference genome database (http://www.wheatgome.org/*).*

## Results

### Phenotypic variation of SOD activities in wheat grains

The SOD activities of Berkut ranged from 1817.49 to 1843.44 U·g^− 1^ across four environments, whereas those of Worrakatta ranged from 1698.44 to 1711.11 U·g^− 1^ (Table 1). The mean SOD activities of RILs in four environments were between 1746.99 U·g^− 1^~1785.97 U·g^− 1^ (Table 1), exhibiting a normal distribution in the population (Fig. S1). The SOD activities showed significant correlations (*P* < 0.01) among different environments (Table 1). The coefficients of variation in the RIL population ranged from 3.9 to 7.1% across environments. The broad-sense heritability based on the mean SOD activity was 0.8.

**Table 1:** Statistical analysis of SOD activities in wheat grains in the RIL population of ‘Berkut’ $\times$ ‘Worrakatta’

	Parent					*CV*				
Environment	B (U·g^-1^)	W (U·g^-1^)	Mean (U·g^-1^)±SD	Range (U·g^-1^)	*CV*	E1	E2	E3	E4	*H* ^2^
E1	1843.44	1710.93	1785.97 ± 126.22	1248.07-2237.51	7.07^**^					0.75
E2	1817.49	1711.11	1752.78 ± 77.27	1528.67-2119.46	4.41^**^	0.65^**^				
E3	1828.77	1698.44	1749.13 ± 79.16	1492.51-2104.03	4.50^**^	0.38^**^	0.51^**^			
E4	1822.85	1708.44	1746.99 ± 72.20	1419.53-2136.27	4.13^**^	0.46^**^	0.48^**^	0.52^**^		
A	1828.14	1707.23	1758.33 ± 68.25	1490.18-2126.42	3.88^**^	0.85^**^	0.83^**^	0.71^**^	0.74^**^	

E1: 2016; E2: 2017; E3: 2018; E4: 2019; A: average value; **Significant at *P*<0.01; SD: Standard deviation; *CV*: Coefficients of variation; B: Berkut; W: Worrakatta *H*^2^: broad-sense heritability

### QTL for SOD activity

**Fig. 1:**
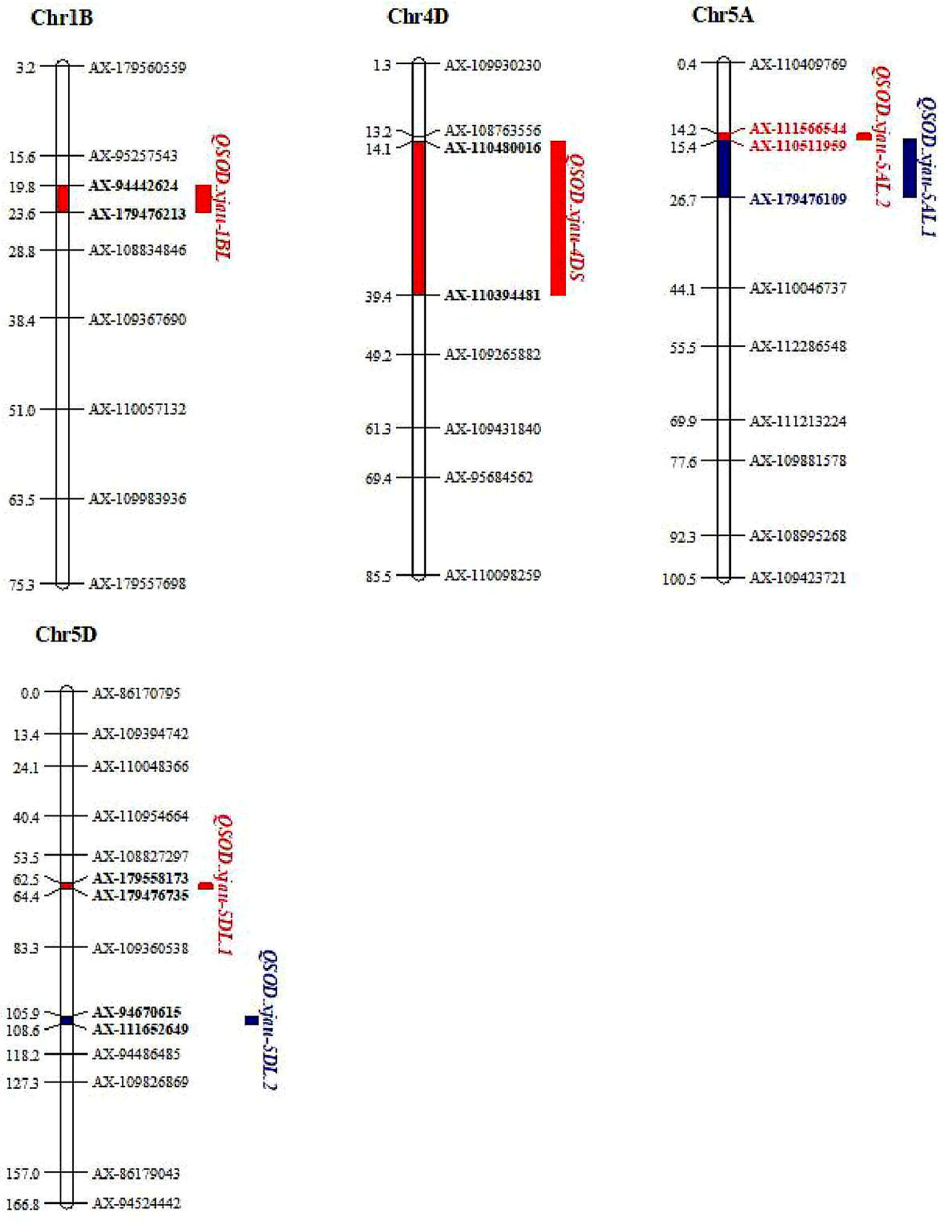
QTL for wheat grain SOD activity identified in the RIL population of ‘Berkut’ × ’Worrakatta’. Note: Markers and QTL are on the right side of the chromosomes, and the genetic positions of the QTL are on the left. The environments where the QTL were detected are shown in different colors (proximal region of linkage groups in red color, the distal region of linkage groups in blue color) on the right of the linkage groups. The flanking markers of QTL are highlighted in bold

Six QTL for SOD activity were detected on chromosomes 1B, 4D, 5 A (2), and 5D (2), designated *QSOD.xjau-1BL*, *QSOD.xjau-4DS*, *QSOD.xjau-5 A.1*, *QSOD.xjau-5 A.2*, *QSOD.xjau-5DL.1*, and *QSOD.xjau-5DL.2* (Fig. 1), respectively, explaining 2.2 to 7.4% of the phenotypic variances (Table 2). The favorable alleles for increasing SOD activity at *QSOD.xjau-4DS* and *QSOD.xjau-5DL.2* loci, were derived from the parental line Berkut, whereas those of *QSOD.xjau-1BL*, *QSOD.xjau-5AL.1*, *QSOD.xjau-5AL.2* and *QSOD.xjau-5DL.1* came from another parental line Worrakatta. *QSOD.xjau-5AL.2* and *QSOD.xjau-5DL.2* were identified in E2 environment and the averaged value of four environments in the RIL population. *QSOD.xjau-5AL.2* was mapped on chromosome 5AL in the region of 577.69 ~ 579.98 Mb, flanked by markers *AX-111566544* and *AX-110511959*, explaining 7.1 ~ 7.4% of the phenotypic variances. *QSOD.xjau-5DL.2* was located between markers *AX-94670615* and *AX-111652649* in the 473.90 ~ 481.42 Mb region of chromosome 5DL, accounting for 2.6 ~ 4.0% of the phenotypic variances.

**Table 2:** Locations and effects of QTL for SOD activity detected in the RIL population of ‘Berkut’ $\times$ ‘Worrakatta’

QTL	Marker Interval	Position (Mb)	LOD	PVE (%)	Add	Environment
*QSOD.xjau-1BL*	*AX-94442624 -AX-179476213*	665.80~668.96	2.06	2.92	13.39	E3
*QSOD.xjau-4DS*	*AX-110480016 -AX-110394481*	8.01~28.05	2.22	2.20	-13.74	E2
*QSOD.xjau-5AL.1*	*AX-110511959 -AX-179476109*	567.51~577.69	2.86	4.99	25.04	E1
*QSOD.xjau-5AL.2*	*AX-111566544 -AX-110511959*	577.69~579.98	7.97	7.39	25.42	E2
	*AX-111566544-AX-110511959*	577.69~579.98	5.96	7.12	20.18	A
*QSOD.xjau-5DL.1*	*AX-179558173-AX-179476735*	393.06~400.88	2.10	2.32	11.40	A
*QSOD.xjau-5DL.2*	*AX-94670615 -AX-111652649*	473.90~481.42	2.75	2.59	-14.92	E2
	*AX-94670615-AX-111652649*	473.90~481.42	3.42	3.96	-14.92	A

E1: 2016; E2: 2017; E3: 2018; E4: 2019; A: average value; LOD: logarithm of odds; PVE: phenotypic variance explained. The physical positions of QTL are based on the Chinese Spring (CS) RefSeq v2.1

### Candidate genes

Four candidate genes for *QSOD.xjau-4DS*, *QSOD.xjau-5AL.1*, and *QSOD.xjau-5DL.1* (2), were identified based on the significant SNPs associated with SOD activity (Table 3). These candidate genes are involved in the SOD metabolism or regulation pathways. *TraesCS4D01G059500* for *QSOD.xjau-4DS* was predicted to be the third type of mammalian superoxide dismutase, an extracellular Cu and Zn superoxide dismutase, referred to as SOD3. *TraesCS5A01G371600* and *TraesCS5D01G343100LC* in *QSOD.xjau-5AL.1* and *QSOD.xjau-5DL.1* loci, respectively, were found to be superoxide dismutase [Cu-Zn], an important enzyme in cellular oxygen metabolism. *TraesCS5D01G299900* for *QSOD.xjau-5DL.1*, encodes an Ethylene-responsive transcription factor, related with SOD activity in plant salinity tolerance.

**Table 3:** Candidate genes predicted in the QTL regions

QTL	Marker interval	Position (Mb)	Gene	Gene annotation
*QSOD.xjau-4DS*	*AX-110480016 -AX-110394481*	35.17	*TraesCS4D01G059500*	Superoxide dismutase [Cu-Zn] 3
*QSOD.xjau-5AL.1*	*AX-110511959 -AX-179476109*	570.37	*TraesCS5A01G371600*	Superoxide dismutase [Cu-Zn]
*QSOD.xjau-5DL.1*	*AX-179558173-AX-179476735*	371.12	*TraesCS5D01G343100LC*	Superoxide dismutase [Cu-Zn]
		397.09	*TraesCS5D01G299900*	Ethylene-responsive transcription factor

The physical locations of QTL are based on the Chinese Spring (CS) RefSeq v2.1, and these candidate genes are annotated in the Chinese Spring (CS) RefSeq v2.1

## Discussion

### Determination of SOD activity

Superoxide dismutase is a key enzyme involved in cellular defense against reactive oxygen species in living organisms, and is widely distributed in microorgnisms, plant and animals [18]. Wang et al. [13] reported SOD activity of 1316.83 ~ 2025.14 U·g^− 1^ in wheat grains, and the coefficient of variations in different environments ranged from 4.3 to 5.2%, indicating that different materials exhibit rich variations of SOD activity among environments. Therefore, accurate determination of SOD activity in wheat grains is important for discovering genes or genetic loci in the wheat genome. The methods for determining SOD activities were described previously, and one method based on SOD matrix -O_2_- needs an expensive equipment and is not stable [19]. In contrast, indirect assays of SOD activities are frequently used, e.g., photoreduction method based on the NBT reduction [20], pyrogallol autoxidation [21], Cytochrome C reduction method [22] and xanthene / xanthene oxidase (XOD) system [23]. The NBT method is widely used for SOD determination due to its low cost, small sample volume, high degree of automation, and convenient procedure [24]. Particularly, the determination of superoxide anion radical (o^2^-) and SOD activity using NBT photoreduction are performed mostly in both plants and animals [25, 26]. Whereas, the NBT method has drawback of a relatively poor soluble formazan, and its accuracy is also affected by the protein content in the reaction system, however, the catalase, glutathione peroxidase and co-enzyme Q10 did not interfere with SOD activity determination in NBT assay [24]. In low temperature and low pH, SOD activity was stable, the crude enzyme solution has higher activity [27].

### QTL associated with SOD activity in wheat

The SOD activities in crops are conditioned by polygenes [11], exhibiting significant variations among environments [28, 29], and the high variations of SOD activities in the stress due to different sensitivities of lines to stress [30], which is in agreement with the present study. And SOD activities are widely employed to indicate plant seed germination [31, 32], premature senescence [33], water stress [34], and micronutrient [35]. Previously, several seed SOD QTL were identified in wheat on chromosomes 1 A, 1B, 1D, 2D, 3 A, 4 A, 7 A [36]. Here, QTL *QSOD.xjau-1BL*, *QSOD.xjau-4DS*, *QSOD.xjau-5AL.1*, *QSOD.xjau-5AL.2*, *QSOD.xjau-5DL.1* and *QSOD.xjau-5DL.2* for SOD activities in wheat grain were identified on chromosomes 1B, 4D, 5 A (2) and 5D (2), respectively. Among these, QTL *QSOD.xjau-1BL* was identified on chromosome 1BL at region from 665.80 to 668.96 Mb. An unconditional QTL for SOD activity were detected on chromosome 1B (*swes119a-ubc857a*) in wheat grain during germination [36]. The QTL *qSOD.1B.SH* for SOD activity was detected on chromosome 1B (*xcfd15-xgwm264*) in wheat leaves [36]. The *QSOD.xjau-5DL.1* was located on the interval 393.06 ~ 400.88 Mb of chromosome 5DL, in agreement with Wang et al. [13] who identified a QTL linked with the marker *RAC875_c49940_385* at 399.30 Mb on chromosome 5D in wheat grain by GWAS panel, whereas *QSOD.xjau-5DL.1* was at 6.24 Mb to 1.58 Mb that is much distant from marker *RAC875_c49940_385*. Therefore, *QSOD.xjau-1BL*, *QSOD.xjau-4DS*, *QSOD.xjau-5 A.1*, *QSOD.xjau-5 A.2*, and *QSOD.xjau-5DL.2* identified in the present study are likely to be new loci for SOD activity.

### Candidate genes of QTL

The four candidate genes identified in the present study are involved in regulation of SOD dismutase, including three SOD genes and a gene related with SOD activity. *TraesCS4D01G059500*, *TraesCS5A01G371600* and *TraesCS5D01G343100LC* were predicted to be SOD genes, located on chromosomes 4DS, 5AL and 5DL, respectively. In addition, a Cu / Zn-SOD gene was found to be present in chromosome 4DL (at chromosome region of 405.45 Mb) [12]. *TraesCS5D01G299900* found in *QSOD.xjau-5DL.1* locus encodes Ethylene-responsive transcription factor. In the MAPK signaling pathway, *ERF1b* interacts with *MYC2*, indicating that *ERF1b* may be a key integration factor in ETH/JA dependent defense regulation of ETH/JA signaling [37, 38]. ERF transcription factors and ethylene along with oxygen dependent signal transduction, play an important role in hypoxic responses [39]. Several QTL on chromosomes 5 A and 5D are associated with SOD, and these QTL are stable across environments. Future studies can focus on these two chromosomes to further dissect the genetic mechanism underlying the activity of SOD in wheat grain.

### Wheat lines with high SOD activity

The SOD gene *TaSOD* showed up-regulation or significantly increased expression levels in wheat after drought and salt stresses [12]. The drought-tolerant genotype had significantly higher level of expression for Mn-SOD and Cu/Zn-SOD than susceptible genotype [40]. In the present study, some F_6_ RILs developed from the cross of ‘Berkut’ × ‘Workatta’ had high SOD activities in the population. The lines B104, B113, B109, B223, B224, B230, and B106 have higher level of SOD activity (2126.42 ~ 1911.47 U·g^− 1^) than other RILs. Favorable alleles for six QTL *QSOD.xjau-1BL*, *QSOD.xjau-4DS*, *QSOD.xjau-5AL.1*, *QSOD.xjau-5AL.2*, *QSOD.xjau-5DL.1*, and *QSOD.xjau-5DL.2* contributed to the high SOD activities in these lines (Table S1). The mean SOD activities of RILs harboring six, five, four, three, two and one favorable alleles are 1954.80 U·g^− 1^, 1796.92 U·g^− 1^, 1773.88 U·g^− 1^, 1770.21 U·g^− 1^, 1757.59 U·g^− 1^ and 1737.42 U·g^− 1^ in four environments of SOD activities. The line B104 had the highest SOD activity of 2126.42 U·g^− 1^ possessing six favorable alleles from QTL *QSOD.xjau-1BL*, *QSOD.xjau-4DS*, *QSOD.xjau-5AL.1*, *QSOD.xjau-5AL.2*, *QSOD.xjau-5DL.1* and *QSOD.xjau-5DL.2*; the line B109 containing five favorable alleles from five loci (*QSOD.xjau-1BL*, *QSOD.xjau-5AL.1*, *QSOD.xjau-5AL.2*, *QSOD.xjau-5DL.1* and *QSOD.xjau-5DL.2*) had 1943.16 U·g^− 1^ of SOD activity; and the line B106 with five favorable alleles at loci of *QSOD.xjau-1BL*, *QSOD.xjau-5AL.1*, *QSOD.xjau-5AL.2*, *QSOD.xjau-5DL.1*, and *QSOD.xjau-5DL.2* in had 1911.47 U·g^− 1^ of SOD activity; and four favorable alleles at loci of *QSOD.xjau-1BL*, *QSOD.xjau-5AL.1*, *QSOD.xjau-5DL.1* and *QSOD.xjau-5DL.2* were found in the line B222 that had 1898.94 U·g^− 1^ of SOD activity; and the other lines B104, B109, and B106 also had higher level of SOD activities among the population. The favorable alleles at these loci could be benefited for improvement of SOD activity in wheat breeding, and those lines with favorable alleles of QTL, and high and stable SOD activity can be integrated into wheat germplasm, and be used to improve the abiotic stress tolerance and quality in wheat.

## Conclusion

Six QTL for SOD activity were detected on chromosomes 1BL, 4DS, 5AL (2), and 5DL (2), respectively, in the RIL population of ‘Berkut’ × ‘Worrakatta’, explaining 2.2 ~ 7.4% of the phenotypic variances. Among four candidate genes predicted in the region of QTL, *TraesCS4D01G059500*, *TraesCS5A01G371600*, *TraesCS5D01G343100LC* and *TraesCS5D01G299900* are annotated as superoxide dismutase [Cu-Zn] 3, superoxide dismutase [Cu-Zn], superoxide dismutase [Cu-Zn] and Ethylene-responsive transcription factor, respectively. Seven wheat lines with high SOD activities can be used as germplasms to improve abiotic stress tolerance and quality in wheat breeding. In the future, fine mapping of SOD candidate genes will further elucidate the genetic dissection of SOD activity in wheat grains.

### Electronic supplementary material

Below is the link to the electronic supplementary material.

Supplementary Material 1

Supplementary Material 2

## Data Availability

Data availability statementsThe datasets generated or analysed during this study are included in this article and its Additional file or are available from the corresponding author on reasonable request.
