# Supplementary material for: Identification of quantitative trait loci and candidate genes for grain superoxide dismutase activity in wheat
Source: BMC Plant Biol. 2024 Jul 27;24:716. doi: 10.1186/s12870-024-05367-z (PMC11282854; doi:10.1186/s12870-024-05367-z)
Supplement: Supplementary file 1 — Supplementary Material 1 [file 12870_2024_5367_MOESM1_ESM.docx]

Supplementary files

Fig. S1 Frequency distribution of SOD activities in the RIL population of ‘Berkut’ × ‘Worrakatta’.

E1: 2016; E2: 2017; E3: 2018; E4: 2019; A: average value; Data are the means (U·g^-1^).
