# Supplementary material for: Identification of quantitative trait loci and candidate genes for grain superoxide dismutase activity in wheat
Source: BMC Plant Biol. 2024 Jul 27;24:716. doi: 10.1186/s12870-024-05367-z (PMC11282854; doi:10.1186/s12870-024-05367-z)
Supplement: Supplementary file 2 — Supplementary Material 2 [file 12870_2024_5367_MOESM2_ESM.docx]

**Supplementary Table S1** The favorable alleles of QTL for wheat grain SOD activity in the RIL population of ‘Berkut’ × ‘Worrakatta’

| Line | Mean (U·g^-1^) | *QSOD.xjau-1BL* | *QSOD.xjau-4DS* | *QSOD.xjau-5AL.1* | *QSOD.xjau-5AL.2* | *QSOD.xjau-5DL.1* | *QSOD.xjau-5DL.2* | Number of favorable allele |
| --- | --- | --- | --- | --- | --- | --- | --- | --- |
| B001 | 1697.48 | A | A | A | A | A | H | 5 |
| B002 | 1670.28 | B | H | H | B | A | B | 1 |
| B003 | 1661.41 | A | H | H | B | B | A | 2 |
| B004 | 1702.04 | B | B | A | H | A | A | 3 |
| B005 | 1687.83 | A | B | H | B | A | B | 2 |
| B006 | 1673.76 | B | B | B | B | H | B | 0 |
| B007 | 1692.25 | B | A | B | B | B | B | 1 |
| B008 | 1691.22 | A | B | B | B | H | B | 1 |
| B009 | 1661.05 | A | H | A | A | H | B | 3 |
| B010 | 1682.04 | B | B | B | B | B | A | 1 |
| B011 | 1583.76 | A | H | H | B | B | B | 1 |
| B012 | 1538.14 | B | H | H | B | B | A | 1 |
| B013 | 1665.62 | B | A | B | B | B | B | 1 |
| B014 | 1641.02 | B | B | H | B | B | B | 0 |
| B015 | 1671.34 | B | B | B | B | A | B | 1 |
| B016 | 1631.37 | B | B | B | B | B | B | 0 |
| B017 | 1710.93 | B | B | B | B | B | B | 0 |
| B018 | 1641.69 | B | B | A | A | B | A | 3 |
| B019 | 1670.62 | B | H | B | B | H | B | 0 |
| B020 | 1695.91 | H | H | B | B | A | A | 2 |
| B021 | 1761.90 | A | B | B | B | A | A | 3 |
| B022 | 1705.85 | A | A | B | B | B | B | 2 |
| B023 | 1723.00 | A | H | H | A | H | B | 2 |
| B024 | 1670.31 | H | H | B | B | A | B | 1 |
| B025 | 1667.38 | A | H | B | B | B | B | 1 |
| B026 | 1740.98 | A | H | A | A | B | A | 4 |
| B027 | 1762.14 | H | B | B | B | A | A | 2 |
| B028 | 1767.35 | A | B | A | A | A | A | 5 |
| B029 | 1702.73 | A | A | B | B | A | B | 3 |
| B030 | 1760.45 | B | B | A | A | A | B | 3 |
| B031 | 1750.88 | B | H | A | A | B | H | 2 |
| B032 | 1740.68 | B | A | B | B | B | A | 2 |
| B033 | 1669.79 | H | A | B | B | B | A | 2 |
| B034 | 1490.18 | H | B | H | B | B | B | 0 |
| B035 | 1710.94 | A | H | H | B | B | A | 2 |
| B036 | 1536.83 | B | H | B | B | A | B | 1 |
| B037 | 1508.23 | H | H | B | B | B | A | 1 |
| B038 | 1735.39 | B | B | B | B | H | A | 1 |
| B039 | 1728.03 | B | A | A | A | B | A | 4 |
| B040 | 1719.87 | A | A | B | B | B | A | 3 |
| B041 | 1746.28 | A | H | A | A | A | B | 4 |
| B042 | 1745.40 | B | H | H | A | B | A | 2 |
| B043 | 1686.53 | B | H | A | A | B | A | 3 |
| B044 | 1811.65 | A | B | A | A | A | B | 4 |
| B045 | 1727.66 | A | H | B | B | B | A | 2 |
| B046 | 1789.38 | A | A | H | B | A | A | 4 |
| B047 | 1799.87 | A | B | A | H | H | B | 2 |
| B048 | 1783.18 | A | A | A | A | A | A | 6 |
| B049 | 1783.41 | H | B | B | B | B | A | 1 |
| B050 | 1771.44 | B | A | H | H | A | A | 3 |
| B051 | 1788.34 | H | B | H | H | H | H | 0 |
| B052 | 1707.00 | A | B | H | H | A | B | 2 |
| B053 | 1808.96 | B | H | A | A | A | B | 3 |
| B054 | 1789.28 | H | H | B | B | A | H | 1 |
| B055 | 1762.25 | H | A | H | H | B | H | 1 |
| B056 | 1760.21 | A | H | B | B | H | H | 1 |
| B057 | 1787.46 | H | B | B | B | B | A | 1 |
| B058 | 1748.47 | B | B | B | B | B | A | 1 |
| B058 | 1800.40 | A | A | B | B | B | A | 3 |
| B059 | 1782.34 | A | H | B | B | A | H | 2 |
| B061 | 1802.80 | H | H | H | A | B | H | 1 |
| B062 | 1810.08 | A | H | B | B | B | H | 1 |
| B063 | 1809.36 | B | H | H | A | B | B | 1 |
| B064 | 1761.78 | B | A | H | H | B | A | 2 |
| B065 | 1756.94 | B | H | H | A | A | A | 3 |
| B066 | 1778.31 | H | A | A | A | B | H | 3 |
| B067 | 1816.32 | B | A | B | B | B | B | 1 |
| B068 | 1747.71 | H | H | H | H | H | H | 0 |
| B069 | 1760.87 | B | H | H | A | B | A | 2 |
| B070 | 1821.66 | A | H | H | A | B | B | 2 |
| B071 | 1787.25 | A | A | B | B | B | A | 3 |
| B072 | 1759.37 | A | H | A | A | B | A | 4 |
| B073 | 1755.19 | A | A | B | B | A | B | 3 |
| B074 | 1778.65 | H | H | B | B | H | B | 0 |
| B075 | 1784.37 | B | H | H | B | A | A | 2 |
| B076 | 1769.88 | B | A | H | B | H | B | 1 |
| B077 | 1777.52 | A | B | H | A | B | A | 3 |
| B078 | 1810.56 | A | A | H | H | B | A | 3 |
| B079 | 1787.81 | H | H | A | A | B | H | 2 |
| B080 | 1801.92 | B | B | H | H | A | A | 2 |
| B081 | 1749.53 | H | H | A | A | B | H | 2 |
| B082 | 1794.65 | B | B | H | H | A | B | 1 |
| B083 | 1776.13 | B | A | A | A | B | H | 3 |
| B084 | 1746.79 | H | B | A | A | B | H | 2 |
| B085 | 1731.53 | A | H | A | A | A | B | 4 |
| B086 | 1719.37 | B | H | B | B | A | A | 2 |
| B087 | 1742.95 | A | A | A | A | A | B | 5 |
| B088 | 1706.32 | B | H | B | B | A | A | 2 |
| B089 | 1684.34 | B | B | H | A | B | B | 1 |
| B090 | 1733.92 | H | A | B | B | A | B | 2 |
| B091 | 1715.60 | H | H | B | B | B | A | 1 |
| B092 | 1720.58 | B | A | H | A | A | B | 3 |
| B093 | 1716.59 | A | H | B | B | H | A | 2 |
| B094 | 1702.27 | H | B | B | B | H | B | 0 |
| B095 | 1714.19 | A | B | H | H | B | B | 1 |
| B096 | 1718.86 | B | B | H | A | B | A | 2 |
| B097 | 1691.65 | A | B | H | A | A | B | 3 |
| B098 | 1722.52 | B | B | H | A | H | B | 1 |
| B099 | 1702.27 | A | B | B | B | A | B | 2 |
| B100 | 1722.76 | A | B | H | A | B | A | 3 |
| B101 | 1712.91 | A | H | H | A | H | H | 2 |
| B102 | 1800.00 | B | A | H | H | B | H | 1 |
| B103 | 1848.58 | B | H | B | B | A | A | 2 |
| B104 | 2126.42 | A | A | A | A | A | A | 6 |
| B105 | 1743.23 | A | H | A | A | A | H | 4 |
| B106 | 1911.47 | A | B | A | A | A | A | 5 |
| B107 | 1852.44 | A | H | A | A | B | B | 3 |
| B108 | 1865.35 | A | B | A | A | A | A | 5 |
| B109 | 1943.16 | A | H | A | A | A | A | 5 |
| B110 | 1770.71 | A | H | B | B | B | A | 2 |
| B111 | 1722.65 | A | H | H | H | B | A | 2 |
| B112 | 1841.24 | A | B | H | H | H | H | 1 |
| B113 | 1975.59 | B | A | B | B | B | H | 1 |
| B114 | 1866.91 | A | H | H | A | A | A | 4 |
| B115 | 1740.28 | A | H | A | A | A | B | 4 |
| B116 | 1845.10 | B | A | B | B | B | H | 1 |
| B117 | 1836.00 | H | H | H | H | H | A | 1 |
| B118 | 1790.97 | B | B | H | A | B | B | 1 |
| B119 | 1797.77 | H | B | B | B | B | A | 1 |
| B120 | 1888.55 | A | H | A | A | H | B | 3 |
| B121 | 1848.95 | B | B | H | A | A | B | 2 |
| B122 | 1811.48 | A | H | H | A | A | A | 4 |
| B123 | 1810.04 | A | B | A | A | A | B | 4 |
| B124 | 1705.27 | H | B | A | A | B | B | 2 |
| B125 | 1796.16 | A | H | A | H | B | A | 3 |
| B126 | 1798.64 | B | A | H | A | A | A | 4 |
| B127 | 1795.67 | A | B | H | A | A | B | 3 |
| B128 | 1757.84 | B | H | H | H | H | B | 0 |
| B129 | 1802.15 | A | A | H | B | A | H | 3 |
| B130 | 1822.68 | B | A | A | A | B | A | 4 |
| B131 | 1771.42 | H | H | A | A | A | H | 3 |
| B132 | 1733.33 | B | B | B | B | B | A | 1 |
| B133 | 1680.74 | B | H | H | H | A | A | 2 |
| B134 | 1726.72 | H | H | H | H | A | A | 2 |
| B135 | 1731.63 | H | H | H | B | A | B | 1 |
| B136 | 1795.91 | B | H | A | A | A | B | 3 |
| B137 | 1768.41 | H | H | B | B | B | A | 1 |
| B138 | 1768.72 | B | H | H | H | H | A | 1 |
| B139 | 1779.27 | A | B | A | A | H | B | 3 |
| B140 | 1803.34 | B | H | A | H | A | A | 3 |
| B141 | 1741.04 | A | B | B | B | B | A | 2 |
| B142 | 1711.25 | B | H | A | A | H | B | 2 |
| B143 | 1660.90 | A | B | B | B | B | A | 2 |
| B144 | 1704.79 | B | B | A | A | A | A | 4 |
| B145 | 1701.44 | B | H | A | A | A | A | 4 |
| B146 | 1705.50 | B | B | B | B | A | B | 1 |
| B147 | 1688.12 | A | H | H | H | H | H | 1 |
| B148 | 1679.89 | A | H | B | B | H | B | 1 |
| B149 | 1669.68 | B | H | H | A | B | B | 1 |
| B150 | 1689.10 | B | H | A | A | A | B | 3 |
| B151 | 1709.19 | H | B | H | A | B | B | 1 |
| B152 | 1702.33 | A | B | H | H | H | A | 2 |
| B153 | 1708.29 | H | B | B | B | B | A | 1 |
| B154 | 1741.08 | B | H | A | A | B | A | 3 |
| B155 | 1704.95 | B | A | B | B | A | A | 3 |
| B156 | 1724.75 | B | H | A | A | A | B | 3 |
| B157 | 1666.24 | H | H | A | A | H | H | 2 |
| B158 | 1688.28 | B | H | H | A | B | B | 1 |
| B159 | 1777.75 | B | H | H | B | A | A | 2 |
| B160 | 1700.44 | A | A | A | A | A | H | 5 |
| B161 | 1705.55 | B | H | B | B | B | A | 1 |
| B162 | 1723.18 | A | A | B | B | A | A | 4 |
| B163 | 1733.49 | B | H | A | A | H | B | 2 |
| B164 | 1732.19 | B | H | H | H | A | A | 2 |
| B165 | 1736.41 | B | A | H | H | H | B | 1 |
| B166 | 1702.02 | B | H | H | A | B | B | 1 |
| B167 | 1725.88 | H | B | A | A | B | A | 3 |
| B168 | 1716.24 | H | H | H | A | H | B | 1 |
| B169 | 1659.22 | A | H | H | A | B | B | 2 |
| B170 | 1632.81 | B | H | B | B | B | A | 1 |
| B171 | 1683.07 | A | H | B | B | A | B | 2 |
| B172 | 1640.58 | A | B | H | A | A | B | 3 |
| B173 | 1643.58 | B | B | A | H | A | B | 2 |
| B174 | 1657.21 | B | H | B | B | B | A | 1 |
| B175 | 1677.80 | A | A | B | B | A | A | 4 |
| B176 | 1700.95 | B | B | B | B | B | B | 0 |
| B177 | 1692.06 | A | H | A | A | B | B | 3 |
| B178 | 1715.66 | B | H | H | A | B | A | 2 |
| B179 | 1730.96 | B | B | B | B | H | B | 0 |
| B180 | 1722.28 | A | H | B | B | B | A | 2 |
| B181 | 1767.75 | H | H | H | H | H | H | 0 |
| B182 | 1804.78 | A | B | A | A | B | H | 3 |
| B183 | 1747.98 | A | B | H | A | A | A | 4 |
| B184 | 1754.74 | A | A | A | A | B | B | 4 |
| B185 | 1796.33 | A | A | H | A | B | A | 4 |
| B186 | 1790.36 | A | A | H | B | H | A | 3 |
| B187 | 1781.69 | B | H | H | A | B | B | 1 |
| B188 | 1790.96 | B | A | B | B | A | A | 3 |
| B189 | 1779.70 | A | H | H | H | H | A | 2 |
| B190 | 1734.85 | B | H | A | A | H | H | 2 |
| B191 | 1790.78 | B | A | B | B | B | A | 2 |
| B192 | 1775.11 | A | A | H | A | A | B | 4 |
| B193 | 1749.23 | H | A | H | H | A | A | 3 |
| B194 | 1800.32 | B | A | H | A | A | B | 3 |
| B195 | 1787.05 | H | H | H | A | A | H | 2 |
| B196 | 1771.37 | A | B | A | A | A | H | 4 |
| B197 | 1800.36 | B | B | H | A | A | B | 2 |
| B198 | 1758.15 | H | A | A | A | B | H | 3 |
| B199 | 1774.63 | H | H | B | H | H | A | 1 |
| B200 | 1745.71 | B | B | H | A | A | B | 2 |
| B201 | 1748.19 | H | A | B | B | A | A | 3 |
| B202 | 1809.65 | B | A | A | A | A | A | 5 |
| B203 | 1739.52 | B | H | H | H | H | A | 1 |
| B204 | 1759.60 | A | B | H | A | A | A | 4 |
| B205 | 1745.35 | B | H | H | A | H | A | 2 |
| B206 | 1741.44 | A | A | B | B | A | A | 4 |
| B207 | 1768.86 | A | H | H | B | A | A | 3 |
| B208 | 1759.54 | A | H | B | B | A | A | 3 |
| B209 | 1777.97 | B | A | A | A | B | A | 4 |
| B210 | 1746.64 | B | H | B | B | A | H | 1 |
| B211 | 1734.16 | B | H | A | A | B | A | 3 |
| B212 | 1753.72 | B | B | A | A | A | B | 3 |
| B213 | 1756.57 | A | B | B | B | B | A | 2 |
| B214 | 1739.28 | H | H | A | A | B | H | 2 |
| B215 | 1748.15 | A | A | H | A | H | A | 4 |
| B216 | 1733.06 | H | H | A | H | H | A | 2 |
| B217 | 1776.36 | B | A | H | H | A | A | 3 |
| B218 | 1717.54 | B | A | A | A | H | B | 3 |
| B219 | 1774.73 | A | H | H | B | H | B | 1 |
| B220 | 1758.32 | B | A | B | B | H | H | 1 |
| B221 | 1837.21 | B | A | H | A | B | B | 2 |
| B222 | 1898.94 | A | B | A | H | A | A | 4 |
| B223 | 1937.62 | B | B | B | B | A | A | 2 |
| B224 | 1935.65 | B | H | A | A | A | B | 3 |
| B225 | 1971.49 | H | H | H | H | A | A | 2 |
| B226 | 1950.09 | H | A | H | H | A | H | 2 |
| B227 | 1888.48 | A | B | H | B | H | A | 2 |
| B228 | 1798.04 | B | A | A | A | H | B | 3 |
| B229 | 1806.55 | B | A | A | A | B | H | 3 |
| B230 | 1915.32 | A | B | B | B | A | A | 3 |
| B231 | 1875.91 | A | B | A | A | A | B | 4 |
| B232 | 1831.48 | H | H | B | B | H | H | 0 |
| B233 | 1782.39 | A | H | A | A | H | B | 3 |
| B234 | 1900.79 | A | H | H | H | B | A | 2 |
| B235 | 1924.90 | A | H | H | A | H | A | 3 |
| B236 | 1806.98 | A | A | B | B | A | B | 3 |
| B237 | 1845.31 | H | H | A | A | B | B | 2 |
| B238 | 1803.99 | B | H | H | A | A | B | 2 |
| B239 | 1815.40 | H | B | B | B | A | A | 2 |
| B240 | 1859.37 | H | H | A | A | A | B | 3 |
| B241 | 1791.43 | A | B | B | B | A | A | 3 |
| B243 | 1776.65 | B | H | H | H | H | B | 0 |
| B244 | 1770.20 | B | H | A | A | A | B | 3 |
| B245 | 1751.17 | B | B | H | H | B | A | 1 |
| B246 | 1784.17 | B | A | H | A | B | B | 2 |
| B247 | 1744.18 | H | B | H | H | H | B | 0 |
| B248 | 1777.72 | A | H | A | A | A | B | 4 |
| B249 | 1779.86 | A | H | H | H | B | A | 2 |
| B250 | 1801.22 | H | A | B | B | A | B | 2 |
| B251 | 1800.32 | B | A | A | A | A | B | 4 |
| B252 | 1722.79 | B | B | B | H | B | A | 1 |
| B253 | 1784.21 | H | H | H | H | B | H | 0 |
| B254 | 1748.90 | A | B | H | A | A | B | 3 |
| B255 | 1762.28 | A | H | H | H | A | B | 2 |
| B256 | 1763.27 | A | H | A | A | B | B | 3 |
| B257 | 1777.86 | H | B | H | B | A | A | 2 |
| B258 | 1775.48 | A | A | A | A | B | H | 4 |
| B259 | 1742.79 | B | B | H | B | B | B | 0 |
| B260 | 1784.99 | H | B | B | B | B | A | 1 |
| B261 | 1755.70 | A | H | A | H | H | A | 3 |
| B262 | 1753.10 | A | A | H | B | B | A | 3 |
| B263 | 1759.31 | H | B | A | H | B | A | 2 |
| B264 | 1776.75 | A | H | A | A | A | B | 4 |
| B265 | 1741.49 | A | H | A | A | A | B | 4 |
| B266 | 1751.10 | A | H | H | H | H | B | 1 |
| B267 | 1777.37 | H | H | H | H | A | B | 1 |
| B268 | 1757.71 | H | A | H | A | B | B | 2 |
| B269 | 1765.56 | A | H | A | A | B | H | 3 |
| B270 | 1788.03 | A | H | A | A | A | B | 4 |
| B271 | 1759.21 | B | A | H | A | B | B | 2 |
| B272 | 1762.07 | A | H | A | H | H | A | 3 |
| B273 | 1770.47 | B | A | H | B | B | A | 2 |
| B274 | 1776.51 | A | B | H | A | B | A | 3 |
| B275 | 1744.85 | B | H | B | B | B | A | 1 |
| B276 | 1733.91 | B | B | A | A | A | A | 4 |
| B277 | 1775.19 | H | A | B | B | B | A | 2 |
| B278 | 1733.49 | H | H | H | B | B | A | 1 |
| B279 | 1765.13 | B | H | B | B | B | A | 1 |
| B280 | 1745.89 | A | B | B | B | B | A | 2 |
| B281 | 1812.06 | H | B | H | H | H | A | 1 |
| B282 | 1772.98 | B | B | H | A | H | B | 1 |
| B283 | 1782.28 | H | H | A | H | B | B | 1 |
| B284 | 1792.70 | A | B | A | A | H | A | 4 |
| B285 | 1776.02 | A | A | A | A | A | H | 5 |
| B286 | 1819.95 | A | A | B | B | B | A | 3 |
| B287 | 1799.88 | A | A | H | A | H | H | 3 |
| B288 | 1761.98 | A | B | B | B | A | A | 3 |
| B289 | 1759.67 | A | A | H | B | B | A | 3 |
| B290 | 1781.03 | A | A | A | A | H | A | 5 |
| B291 | 1788.24 | A | A | H | H | H | A | 3 |
| B292 | 1755.85 | B | H | H | H | H | H | 0 |
| B293 | 1779.45 | B | H | A | A | B | B | 2 |
| B294 | 1806.02 | B | H | A | A | A | B | 3 |
| B295 | 1780.69 | A | B | A | A | H | A | 4 |
| B296 | 1790.98 | B | B | A | A | A | B | 3 |
| B297 | 1785.95 | A | A | A | H | H | B | 3 |
| B298 | 1820.51 | H | A | B | B | B | H | 1 |
| B299 | 1810.55 | A | A | A | H | A | B | 4 |
| B300 | 1822.38 | B | A | A | A | A | B | 4 |
| B301 | 1738.58 | B | B | H | B | A | A | 2 |
| B302 | 1787.67 | B | H | H | H | A | A | 2 |
| B303 | 1809.28 | H | H | H | B | B | H | 0 |
| B304 | 1771.25 | A | A | A | A | A | B | 5 |
| B305 | 1777.81 | A | H | H | B | A | A | 3 |
| B306 | 1747.00 | H | A | B | B | B | A | 2 |
| B307 | 1750.29 | H | A | A | A | H | H | 3 |
| B308 | 1746.96 | H | H | B | B | B | A | 1 |
| B309 | 1787.35 | A | B | B | B | H | H | 1 |
| B310 | 1769.16 | A | B | B | B | H | B | 1 |
